# Multiplex cytokine profile from dengue patients: MIP-1beta and IFN-gamma as predictive factors for severity

**DOI:** 10.1186/1471-2334-8-86

**Published:** 2008-06-25

**Authors:** Fernando A Bozza, Oswaldo G Cruz, Sonia MO Zagne, Elzinandes L Azeredo, Rita MR Nogueira, Edson F Assis, Patricia T Bozza, Claire F Kubelka

**Affiliations:** 1 Instituto de Pesquisa Clínica Evandro Chagas, Fundação Oswaldo Cruz, Rio de Janeiro, Brazil; 2 Programa de Computação Científica, Fundação Oswaldo Cruz, Rio de Janeiro, Brazil; 3 Hospital Universitário Antonio Pedro, Universidade Federal Fluminense, Niterói, Brazil; 4 Laboratório de Imunologia Viral, Instituto Oswaldo Cruz, Fundação Oswaldo Cruz, Rio de Janeiro, Brazil; 5 Laboratório de Flavivirus, Instituto Oswaldo Cruz, Fundação Oswaldo Cruz, Rio de Janeiro, Brazil; 6 Laboratório de Imunofarmacologia, Instituto Oswaldo Cruz, Fundação Oswaldo Cruz, Rio de Janeiro, Brazil

## Abstract

**Background:**

Dengue virus pathogenesis is not yet fully understood and the identification of patients at high risk for developing severe disease forms is still a great challenge in dengue patient care. During the present study, we evaluated prospectively the potential of cytokines present in plasma from patients with dengue in stratifying disease severity.

**Methods:**

Seventeen-cytokine multiplex fluorescent microbead immunoassay was used for the simultaneous detection in 59 dengue patients. GLM models using bimodal or Gaussian family were determined in order to associate cytokines with clinical manifestations and laboratory diagnosis.

**Results:**

IL-1β, IFN-γ, IL-4, IL-6, IL-13, IL-7 and GM-CSF were significantly increased in patients with severe clinical manifestations (severe dengue) when compared to mild disease forms (mild dengue). In contrast, increased MIP-1β levels were observed in patients with mild dengue. MIP-1β was also associated with CD56+NK cell circulating rates. IL-1β, IL-8, TNF-α and MCP-1 were associated with marked thrombocytopenia. Increased MCP-1 and GM-CSF levels correlated with hypotension. Moreover, MIP-1β and IFN-γ were independently associated with both dengue severity and disease outcome.

**Conclusion:**

Our data demonstrated that the use of a multiple cytokine assay platform was suitable for identifying distinct cytokine profiles associated with the dengue clinical manifestations and severity. MIP-β is indicated for the first time as a good prognostic marker in contrast to IFN-γ that was associated with disease severity.

## Background

During the last decades dengue became the most important arthropod-borne emerging viral disease in tropical countries [1]. It is estimated that about 2.5% notified cases are classified as dengue haemorrhagic fever (DHF) and about 2.5–20% of DHF cases are lethal [2-4]. In the last two decades, Latin America saw a dramatic increase in frequency and in geographic extension of dengue fever. Specifically, the situation in Brazil has worsened during the last decade since the introduction of the Dengue-3 serotype. In the past years Brazil had dengue outbreaks with at least 1 million cases (2001–2002) and within the last 18 months 900 thousand cases were reported [5]. In addition, severe disease forms are occurring with increased frequency and mortality rates.

Dengue pathogenesis is not completely understood, and the main determinants of the development of severe forms are not yet well established. Increase in capillary permeability associated with endothelial activation and haemorrhagic phenomena are landmarks of severe clinical manifestations, strongly suggesting an alteration in immunoregulation [6].

Cytokines are proteins secreted during innate and adaptive immunological responses, acting as inflammatory mediators or modulatory molecules during several haemorrhagic fevers [7]. Clinical studies support a key role for cytokines in the DHF pathogenesis [8-13]. During Dengue virus infections, cytokines are involved in the disease onset and homeostatic regulation. Specifically, TNF-α, IL-1β and IL-6 have been associated with both coagulation (F1+2 and TATc) and fibrinolysis (t-PA, PAPc, and D-dimmer) activation markers [14]. This activation is more striking in patients with severe clinical manifestations, although it can be found at lower degrees in patients with mild disease [15,16].

Despite the fact that cytokine network and their multiple regulatory pathways are highly complex and not fully elucidated during dengue fever, these molecules seem to represent interesting markers for patient stratification or prognosis. An emerging interest has appeared in order to define biomarkers that may have pathophysiological roles during disease and that may be used as future therapeutic targets. New technologies have been developed in order to detect multiple biomarkers within a single and small blood sample. Such approaches may lead to the development of specific marker panels for dengue fever. Accordingly, cytokine patterns have been indicated as serum biomarkers during infectious diseases such as Hepatitis C [17], ARDS [18] and sepsis [19].

In this study, we prospectively evaluated the potential use of plasma cytokine concentrations for severity stratification of patients with dengue, using a 17 cytokine-multiplex assay. Among tested cytokines, we were able to recognize ten significantly altered circulating factors and to characterise cytokine patterns related to determined clinical manifestations and disease severity.

## Methods

### Study population

The Ethics Committee of the Oswaldo Cruz Foundation approved this study protocol and written informed consent was obtained from all patients or their guardians prior to blood collection.

We included prospectively 59 dengue-infected patients (33 females, 26 males, age range 15–73 years) assisted at three Health Centres in Niterói, Rio de Janeiro State (Posto de Saúde de Itaipú, Centro Previdenciário de Niterói and Hospital das Clínicas de Niterói). All patients presented clinical diagnosis of dengue infection according to WHO criteria [3]. Among dengue patients, 39 cases were hospitalised due to severity.

A detailed history and physical examination was performed to detect hemorrhagic manifestations (positive tourniquet test for capillary fragility, skin haemorrhages, epistaxis, gingival, gastrointestinal, or urinary tract haemorrhage), signs of plasma leakage (pleural or pericardial effusion, ascites), signs of circulatory failure (cold extremities, cyanosis, hypotension, tachycardia, shock), and hepatomegaly.

### Case definition

In addition to the suggestive clinical diagnosis, all patients had the Dengue virus infection confirmed either by anti-dengue enzyme-linked immunoabsorbent assay (ELISA)-IgM, serotype specific reverse transcription-polymerase chain reaction (RT-PCR) or by virus isolation [20-22]. Dengue immune response was considered as primary or secondary by IgG ELISA according to previously established criteria [23].

As previously reported [24-26], we also were often unable to characterize the severe disease forms based on WHO criteria [3]. In Nicaragua, Harris *et al. *[24,27] described four key severe clinical manifestations associated with dengue – shock, plasma leakage, marked thrombocytopenia or internal haemorrhage – that do not fit DHF/DSS classification as single parameters. According to these criteria, we considered:

• Severe dengue – Dengue confirmed cases plus severe thrombocytopenia (<50,000 platelets/mm^3^) and/or hypotension (postural hypotension with decrease in systolic arterial pressure in 20 mm Hg in supine position or systolic arterial pressure < 90 mm Hg) and/or plasma leakage (either haemoconcentration fluctuation of packed cell volume ≥ 20% during illness course and recovery or clinical signs of plasma leakage, such as pleural effusion) and/or severe haemorrhagic manifestations.

• Mild dengue – Dengue confirmed cases in absence of severe thrombocytopenia, hypotension, plasma leakage signs or haemorrhagic manifestations.

### Blood samples and cytokine detection by multiplex microbead immunoassay

Blood samples were collected from a peripheral vein and kept on ice. Plasma was collected by centrifugation at 800 g for 15 min at 4°C, aliquoted, and stored at -70°C until the analysis day. A multiplex biometric immunoassay, containing fluorescent dyed microspheres conjugated with a monoclonal antibody specific for a target protein, was used for cytokine measurement according to the manufacturer's instructions (Bio-Plex Human Cytokine Assay; Bio-Rad Inc., Hercules, CA, USA). Cytokines measured were: IL-1*β*, IL-2, IL-4, IL-5, IL-6, IL-7, CXCL8 (IL-8), IL-10, IL-12 (p70), IL-13, IL-17, granulocyte colony stimulating factor (G-CSF), granulocyte-monocyte colony stimulating factor (GM-CSF), monocyte chemoattractive protein (MCP-1/CCL2), macrophage inflammatory protein (MIP-1*β*/CCL4), and TNF-*α*. Briefly, 20 μl plasma samples were diluted 1:4 and incubated with antibody-coupled beads. Complexes were washed, then incubated with biotinylated detection antibody and, finally, with streptavidin-phycoerythrin prior to assessing cytokine concentration titres. Concentrated human recombinant cytokine was provided by the vendor (Bio-Rad Laboratories). A range of 1.95–32,000 pg/ml recombinant cytokines was used to establish standard curves and to maximize the sensitivity and the assay dynamic range. Cytokine levels were determined using a multiplex array reader from Luminex™ Instrumentation System (Bio-Plex Workstation from Bio-Rad Laboratories). The analyte concentration was calculated using software provided by the manufacturer (Bio-Plex Manager Software).

### Extracellular staining for flow cytometry analysis

Liquid nitrogen cryopreserved peripheral blood mononuclear leukocytes were isolated by Histopaque-1077 (Sigma Chemical Co., Saint Louis, MO, USA) from 35 out of 59 dengue patients. Cells were stained for CD56 surface marker using anti-CD56-Cy5 (IgG1, clone B159) from Pharmingen (San Diego, CA, USA) and positive cells were detected by flow cytometry as described before [22] using FACScalibur (Becton-Dickinson). Events (10,000–20,000) were acquired and analyses were carried out with FlowJo (TreeStar, version 4.3) software.

### Statistical analyses

The nonparametric Mann-Whitney U test was used to evaluate differences between cytokine ratios from severe and mild dengue patients.

GLM models were used to evaluate factors independently associated with quantitative variables. Analysis of factors independently associated with dengue severity and other clinical manifestations was performed with GLM with logistic regression or Gaussian family. Results from the logistic regressions are given as odds ratio (OR). The confidence interval (CI) was established at 95%. Alternatively, for a GLM Gaussian family t values were recorded. A probability value of *P*<0.05 was considered to be significant. The statistical programs R [28] and Prism 4 (GraphPad Software, San Diego, CA, USA) were employed.

The Fisher's exact test was applied to determine the significance of positive samples from patients when comparing different virus serotypes or sequential infections. Correlation between platelet counts and cytokine production in blood samples was estimated by Spearman's correlation.

## Results

### Clinical characterisation of dengue disease in adult Brazilian patients

**Figure 1:**
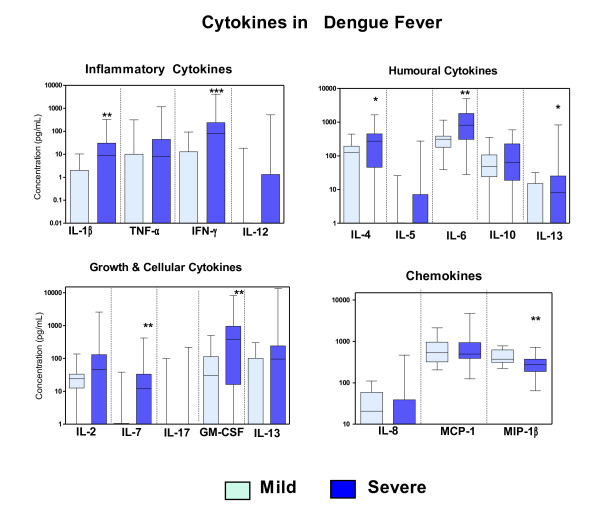
**Cytokine levels in plasma from patients with mild and severe dengue**. Box-and-whiskers graph. The box extends from the 25^th ^to the 75^th ^percentile and the line at the middle is the median. The error bars, or whiskers extend down to the lowest value and up to the highest. Factors were sorted according to their functional groups. Mann-Whitney U test was used to evaluate differences between cytokine concentration from severe and mild dengue patients. * P < 0.05, ** P < 0.01 and ** P < 0.001.

From the 59 patients included, 39 were classified as severe dengue and 20 as mild dengue. Detailed demographic, clinical, and laboratorial data from dengue patients are summarized in Table 1. Blood collection was performed between 3 and 10 days after disease onset. In order to avoid effects due to differences in the blood collection time, we compared mild and severe dengue patient groups using Mann-Whitney U Test, which showed no differences in the disease onset time at the moment of sample collection [see Additional file 1]. The original data used to perform this analysis is shown at Figure 1.

**Table 1:** Demographic information about the study population with dengue fever.

**Characteristics**	**Mild^1^**		**Severe**		**Fisher's exact test**
Age (median years, 25–75%) ^**2**^	39, 28–48		37, 23–53		
Sex (M:F) (Patient Number)	7:13		26:13		
DENV-1: DENV-3 (Patient Number)	20:0		8:31		p < 0.0001
			
	**%**^**3**^	***N***^**4**^	**%**	***N***	

Fever	**30**	*20*	**8**	*37*	
Haemorrhagic manifestations^**5**^	**35**	*20*	**41**	*37*	
Petechia	**21**	*19*	**49**	*37*	
Abdominal Pain	**19**	*16*	**33**	*36*	
Thrombocytopenia (<100.000 counts/mm^3^)	**63**	*19*	**72**	*39*	
Thrombocytopenia (<50.000 counts/mm^3^)	**0**	*19*	**46**	*39*	
Hipotension^**6**^	**0**	*20*	**67**	*39*	
Hemoconcentration^**7**^	**33**	*15*	**10**	*39*	
Secondary Infection	**26**	*19*	**42**	*33*	P = 0.37

^**1 **^See Methods for mild and severe dengue definition.

^**2 **^Study population with 59 patients with 3–10 days of disease onset.

^**3 **^Percentage of positive patients for the measured parameter within the group.

^**4 **^Number of patients used for the measured parameter within the group.

^**5 **^Epistaxis, gengivorrhagia, positive tourniquet test, metrorrhagia, bleeding after coughing.

^**6**^Postural hypotension with decrease in systolic arterial pressure in 20 mmHg in supine position or systolic arterial pressure < 90 mmHg.

^**7 **^Elevated hematocrit for matched age and sex (women>41%; men >45%) or fluctuation of packed cell volume ≥ 20% during course of illness and recovery.

Patients with mild and severe dengue were investigated for prior incidence of infection, detected by serologic immune response (IgG antibodies for DENV). Patients with severe dengue (42%; 14 out 33) were more likely to be experiencing a secondary Dengue virus infection than patients with mild dengue (28%; 6 out 20), although no statistical significance was found in Fisher's exact test (*P *= 0.3989). Among 22 patients with DENV-1, 5 were classified as secondary infection, whereas among 35 patients with DENV-3, 12 were classified as secondary infection (*P *= 0.3912).

### Circulating cytokines are elevated in dengue patients during early febrile phase increasing their levels during defervescence

Dengue fever is characterised by a high fever phase and an abrupt drop in body temperature that has been called defervescence phase. Characteristically the disease outcome is defined during this phase, when patients can either recover rapidly or progress to a severe life-threatening stage. Cytokines and immunoactivation markers such as IFN-γ, IL-2, soluble CD8 and receptors for TNF-α [12,29] are associated with the defervescence phase and with disease severity. IFN-α levels are higher in DHF than in DF during defervescence [30].

During the febrile phase significant increase in cytokine circulating levels was detected including IL-4, IL-6, IL-10, MCP-1 and MIP-1β levels, which were maintained also elevated in defervescence (data not shown, analysed by non parametric Kruskal-Wallis test and Dunn's Multiple Comparison Test when compared with controls, P < 0.05); no significantly altered febrile levels were found when compared to defervescence. During the febrile phase IL-7 was significantly higher than in defervescence. IL-1β, IL-13, IFN-γ were significantly increased during defervescence as compared to control samples. Significant levels of IL-5, IL-12, and IL-17 were not detected during dengue disease in our patients. IL-2 was detected both in healthy individuals and in dengue patients but no difference between these two groups was detected [see Additional file 2].

### Plasma cytokine levels are changed in patients with severe dengue and other clinical manifestations

We studied the cytokine profile from Brazilian patients in order to compare severe and mild dengue cases during the acute phase of the disease. Figure 1 shows data from patients with regard to their plasma cytokine contents, which were sorted in four groups according to their reported function. We observed that cytokine concentrations of IL-1β, IFN-γ, IL-4, IL-6, IL-7, IL-13 and GM-CSF were significantly increased during severe dengue as compared to mild dengue, while MIP-1β levels are higher in mild dengue.

**Figure 2:**
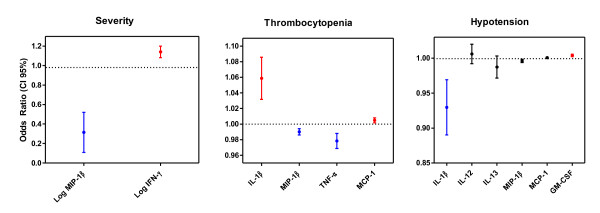
**Cytokines detected in plasma as independent factors in predicting severity**. Normal GLM model originated from logistic regression analysis using binomial family odds ratios (OD) and 95% confidence intervals (CI). Fifty-nine patients with dengue were grouped according with their clinical manifestations: Severe (N = 39), hypotension (N = 24) or thrombocytopenia (N = 18). Details on Table 2.

MIP-1β and IFN-γ were independent variables associated disease outcome as determined by a logistic regression model (Table 2 and Figure 2). While MIP-1β was increased during mild dengue with odds ratio (OR) of 0.181 and confidence interval (CI) 0.045–0.72, IFN-γ was associated with severity with OR of 1.138 (CI, 1.0541–1.245).

**Table 2:** Logistic models with logit links for different clinical manifestations and laboratorial assays during dengue fever based on cytokine production.

**Severity^1 ^**Yes = 39, No = 20 ^3^	**Coefficient**	**Error**	**P value **(significance)^**4**^	**OR**^**2 **^(CI 95% range)
**Intercept**	**10.855**	**4.150**	0.0089**	-
**Log (MIP-1β +1e-4) -**	**1.712**	**0.705**	0.0153*	**0.181**(0.045–0.72)
**Log (IFN-γ +1e-4)**	**0.130**	**0.046**	0.0046**	**1.138 **(1.041–1.245)

**Platelet counts 50.000/mm^3 ^**Yes = 18 No = 40	**Coefficient**	**Error**	**P value **(significance)	**OR **(CI 95% range)

**Intercept**	**0.940**	**0.943**	**0.3187**	-
**MIP-1β**	**-0.010**	**0.023**	0.0057**	**0.990 **(0.983–0.997)
**IL-1β **	**0.056**	**0.023**	0.0123*	**1.058 **(1.012–1.106)
**TNF-α **	**-0.022**	**0.008**	0.0100*	**0.978**(0.962–0.995)
**MCP-1**	**0.002**	**0.001**	0.0437*	**1.002 **(1.0001–1.0036)

**Hypotension **Yes = 24 No = 35	**Coefficient**	**Error**	**P value **(significance)	**OR **(CI 95% range)

**Intercept**	**0.357**	**0.742**	**0.6302**	-
**IL-1β **	**0.075**	**0.038**	0.0454*	**0.928 **(0.862–0.999)
**IL-12**	**0.005**	**0.012**	**0.6516**	**1.006 **(0.982–1.030)
**IL-13**	**-0.013**	**0.014**	**0.3519**	**0.987 **(0.960–1.015)
**MIP-1β **	**-0.004**	**0.002**	**0.0807**	**0.996 **(0.992–1.000)
**MCP-1**	**0.001**	**0.0007**	**0.2065**	**1.001 **(0.9995–1.002)
**GM-CSF**	**0.0043**	**0.0017**	0.0214*	**1.004 **(1.001–1.007)

^**1**^Models were chosen according to their best Akaike information criterion (AIC). In this model we used MIP-1β and IFN-γ log values in order to obtain a small AIC.

^**2**^**OD, **odds ratio; **CI**, confidence interval.

^**3**^Number of patients.

^4 ^* P < 0.05, ** P < 0.01 and *** P < 0.001.

To assess relationships between cytokine levels and several clinical manifestations, the patient cohort with severe dengue was divided into distinct subgroups: those with hypotension, thrombocytopenia (≤50.000 counts/mm^3^) and/or haemorrhagic manifestations. A logistic regression model was used for binomial response subgroups and GLM models using Gaussian family were employed for subgroups with continuous response in order to determine cytokine profiles.

IL-1β was associated with marked thrombocytopenia with OR = 1.058 (CI, 1.012–1.106) in dengue patients. TNF-α was inversely related to thrombocytopenia with OR = 0.978 (CI, 0.962–0.995) (Table 2, Figure 2). Considering platelet counts as a continuous variable for statistical analysis with a Gaussian family, it was possible to determine that IL-8 (P = 0.0434) and MCP-1 (P = 0.0146) levels are inversely related to their counts, displaying therefore an association with thrombocytopenia, while MIP-1β (P = 0.0114) confirms its association with higher counts – normal or tending to normal (Table 3).

**Table 3:** Normal GLM models using Gaussian family for different clinical manifestations and laboratorial assays during dengue fever based on cytokine production.

**MODELS^1^**	**Coefficient**	**Standard Error**	**t value**	**P value (>| t|) (significance)**
**Platelet counts:**				
(N = 59)				
**Intercept**	81.42	18.9963	4.286	8.7e-05 ***^2^
**IL-1**β	-0.180	0.1803	-0.998	**0.3233**
**IL-8**	-0.194	0.094	-2.075	0.0434 *
**IL-13**	0.173	0.081	2.145	0.0371 *
**MIP-1**β	0.130	0.049	2.630	0.0114 *
**MCP-1**	-0.033	0.013	-2.534	0.0146 *

**Hematocrit:**				
(N = 59)				
**Intercept**	38.850	0.867	44.813	<2e-16 ***
**Sex**	4.850	1.043	4.649	2.55e-05 ***
**IL-1**β	0.026	0.012	2.105	0.0405 *
**IL-6**	-0.002	0.0007	-3.074	0.0035 **
**IL-12**	0.011	0.004	2.492	0.0162 *

^**1**^. Models were chosen according to their best Akaike information criterion (AIC).

^**2**^. * P < 0.05, ** P < 0.01 and *** P < 0.001.

GM-CSF (OR = 1.004; CI, 1.001–1.007) was related with hypotension, whereas IL-1β had a negative predictive value (OR = 0.928; CI, 0862–0.999) and MIP-1β had a tendency for a good prognostic (OR = 0.996; CI, 0.992–1.000) as shown in Table 2 and Figure 2.

Natural Killer (NK) cells have been earlier related to mild cases of dengue [22]. Forty-eight PBMC samples from thirty-five patients had their CD56+ rates determined by flow cytometry and a good correlation was observed with their respective MIP-1β plasmatic levels (*r *= 0.4668; *P *= 0.0008).

Considering that different cytokines act in the immunological network as stimulating/up regulating factors and also in a feedback loop as down regulators, the cytokine balance might play a role in the immune response outcome. Therefore we calculated MIP-1β/IFN-γ ratios for every patient and compared those from mild dengue with those from severe dengue. Ratio averages ± SEM were respectively 3032 ± 514 and 864 ± 240 (*P *= 0.0003; Mann Whitney U test), confirming our earlier data that these cytokines are acting as opposing factors. The different models built here using clinical manifestations as independent variables each exhibit specific cytokine profiles.

## Discussion

The cytokine profile identified in patients with dengue may represent a valuable tool for the characterisation of immunological response patterns and may assist the identification of patient groups at risk for developing severe disease. In the present study, the use of a multiplex analysis for cytokine plasma detection in patients with dengue could identify cytokine profiles associated with the disease severity.

Early identification and management of severe dengue disease are essential to prevent death. It has been increasingly recognized that the inflammatory response and deregulated cytokine production play key roles in the development of severe clinical manifestations [31]. However, cytokine profiles associated with dengue evolution and prognosis are not well established. New technologies for cytokine quantification were developed including the multiplex immunofluorescent bead array analysis system, allowing multiple biomarkers to be tested simultaneously in a small volume from one single plasma aliquot. Recently, this methodology has been used for cytokine profile evaluation during several infectious diseases including viral infections [17,32,33] and sepsis [19], among others.

We were able to identify models for cytokine circulation during dengue acute phase that may vary with clinical manifestations. MIP-1β was for the first time associated with a good prognostic and was identified in the different disease models presented here. MIP-1β has been earlier detected after Dengue virus cell stimuli *in vitro *[34,35] but preliminary studies *in vivo *did not report their role in severity. In accordance with a protective role for MIP-1β, changes in MIP-1β levels were significantly correlated with decreases in viral titre after Hepatitis C treatment [17]. In addition, MIP-1β was up regulated in acute infection in chimpanzees only when viral clearance took place, but not in those animals which failed to eradicate the virus [36].

MIP-1β is produced by human monocytes and dendritic cells upon different stimuli [37] as well as by activated NK cells [38] and lymphocytes [39,40]. Activated NK cells release granzyme A, which displays cytolytic functions and MIP-1β is chemoattractant for NK cells, recruiting them to inflammatory sites. NK cells have been associated with mild dengue [22]. Here a good correlation between MIP-1β plasma levels and NK cells was observed, reinforcing the relevance of these pathways and strongly suggesting their role in dengue protective mechanisms. An early and efficient virus clearance by direct or indirect NK functions is likely controlling virus replication, restricting intense immunological activation and the dengue immunopathology and therefore favouring a mild dengue disease.

In previous studies, TNF-α has been reported to be associated with severity, mainly during DHF in Brazilian patients [11,13,41]. In the present study, however, this cytokine was not strongly associated with severity. Indeed, other authors also found inconsistency or no difference in TNF-α levels in severe *vs. *mild disease forms [10,42]. We may hypothesize that differences in Dengue virus serotypes or in host immune response such as different TNF-α genetic polymorphisms may explain the disease outcome. In our study from 2001 (Braga et al., 2001), patients were Dengue-2 infected, while in the present study, patients were Dengue-1 and -3 infected. A recent report [43] describes non-significant TNF-α serum levels in adult patients and suggests that the discrepancy may have been caused by a transient TNF-α peak which was not detected at a later time point.

In the present study we observed an association of IFN-γ with disease severity. Indeed, increased IFN-γ concentrations have been detected in dengue patients in a variety of studies [29,44-47]. DHF induced by Dengue-3 was associated with higher viremia early in illness and earlier peak plasma IFN-γ levels; maximum plasma viremia levels correlated with the degree of plasma leakage and thrombocytopenia [45]. However, in a previous study from our group we failed to observe association of IFN-γ with disease severity [44], probably due to the small number of severe patients analyzed or to the Dengue-1 incidence.

IFN-γ is produced during a T-lymphocyte helper response type 1 and may reflect CD8+ T cell activation with production of inflammatory cytokines. High levels of IFN-γ were observed in patients with dengue from Asian and Latin America and were associated with severity [9]. IFN-γ produced by T cells may also activate mononuclear phagocytes (monocytes and dendritic cells), which would produce factors such as TNF-α, tissue factor, and platelet-activating factor, among other mediators. These factors may all participate in platelet and endothelial cell activation, leading to platelet consumption, increase in endothelial permeability, hypotension and ultimately to shock. IFN-γ has also been associated with secondary heterologous Dengue virus infections [47,48] inducing a strong antigenically cross-reactive inflammatory response, probably inefficient in terms of antibody and T-cell specific response. Indeed, we observed earlier in several patients a CD8+T cell activation with HLA-DR+ subset increase that was associated with severity [9].

GM-CSF acts at early differentiation processes at myeloid progenitors or resting monocytes [49]. An additional stimulus may be required to activate monocytes or dendritic cells in order to produce proinflammatory cytokines [50]. GM-CSF was associated with hypotension as well as MCP-1, likely acting both in concert, contrasting with MIP-1β, once more associated with good prognostics. MCP-1 was earlier detected in DHF patients [51] but for the first time we reported clinical and laboratory findings associated with severity.

IL-8 and MCP-1, here associated with thrombocytopenia, are chemokines and may contribute to platelet activation, either by their chemoattractant properties or by their effect on endothelial permeability. Both factors were detected in patients with DHF [51,52]. These cytokines are produced by monocytes after various activation stimuli, such as virus infection, and increase the endothelial permeability by disrupting tight junctions among cells [53].

Despite the fact that our study could identify cytokines with good accuracy for the stratification and/or prognosis of dengue, it has potential limitations. Here we identified cytokines related to dengue severity, but the small sample size represents a shortcoming regarding the generalization of our results. In addition, only one time point was used for the measurement of cytokines, not allowing further insights provided by sequential measurements. Moreover, classification of disease severity has been a matter of debate, especially for adult patients' management and classification. Indeed, the WHO criteria for DHF has failed to identify severe disease, including fatal cases, in adult Latin America population [24,54] (S.M.O. Zagne, R.M.O. Nogueira, unpublished) and clearly do not satisfy the stratification of our studied population. Accordingly, in the present study severe disease forms were classified following other proposed criteria [24]. While a direct correlation of cytokine concentrations and the pathophysiology of severe dengue is tempting, we believe that the full burden of disease severity cannot be attributed to a single cytokine. Cytokines may be increased simply as one of the several steps in the network loops without necessarily playing a direct harmful role and most likely more than one factor may be involved, including others not tested here such as IL-18, TGF-β, and MIF among others [9].

**Figure 3:**
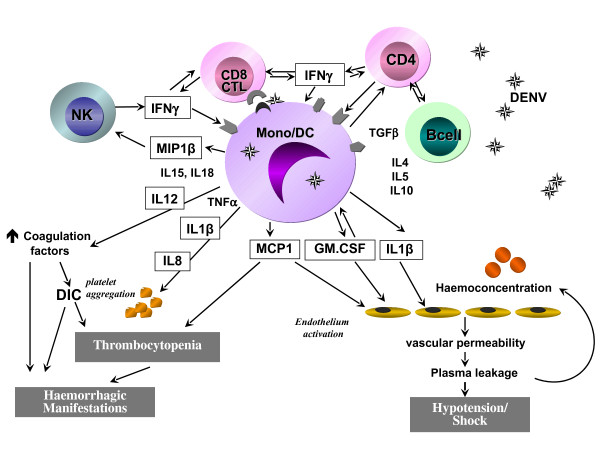
**Hypothetic mechanism to explain cytokine models during dengue fever**. MIP-1β is associated with a good prognostic and IFN-γ has a predictive value for severity. GM-CSF, MCP-1, IL-1β, IL-6, IL-8, IL-12, IL-13 are also playing important roles during dengue pathogenesis (see text for detailed description).

We can suggest a mechanism explaining our cytokine models for dengue fever (Figure 3). MIP-1β would be associated with a protective pathway for its chemoattractive and activating effect on NK cells, which in turn are efficient cells in early virus clearance, by their antiviral cytokine production and cytotoxic activity against infected target cells. IFN-γ has a deleterious effect for the host regarding its action in activating T cells for virus antigenic cross-reactive response and monocyte/dendritic cell activation. Mononuclear monocytes are activated by IFN-γ and GM-CSF among other cytokines and in turn produce several factors such as IL-1β and MCP-1 that may act on vascular permeability leading to plasma leakage and haemoconcentration. As suggested by other authors, it is likely that viral replication in antigen presenting cells, cytokine liberation and circulation, and T cell activation may not be a linear process [55], but in fact a complex interaction network, with positive and negative feedbacks, where viral clearance and pathologic events take place, such as increased vascular permeability and circulatory collapse, and their balance may determine the disease outcome.

## Conclusion

Our study demonstrated the plasma cytokine profile in dengue fever from a Brazilian population detected by a multiplex bead immunoassay. MIP-β is indicated for the first time as a good prognostic marker and in contrast to IFN-γ that was associated with the disease severity. Both cytokines can discriminate mild from severe cases. Moreover, we show here for the first time that during the dengue course different cytokine profiles may be present and vary according to determined clinical manifestations. The cytokine profiles identified herein by bead array multiplex system may favour an early identification of patients with the worst prognosis and may contribute to the establishment of more directed therapeutic procedures than the present ones.

## Competing interests

The authors declare that they have no competing interests.

## Authors' contributions

FAB and OGC contributed equally to the study. FAB contributed to the study conception and design, carried out clinical studies, helped in data analysis and in drafting the manuscript. OGC performed data and statistical analysis. SMOZ carried out the clinical studies. EFA and carried out the Luminex immunoassays. ELA collected and stored samples and patient data and helped in the Luminex immunoassays. RMRN was responsible for the confirmatory diagnostics. PTB conceived the study and design and helped to draft the manuscript. CFK conceived the study and design, participated in data and statistical analysis and drafted the manuscript. All authors read and approved the final manuscript.

## Pre-publication history

The pre-publication history for this paper can be accessed here:



## Supplementary Material

Additional file 1 **Blood collection time in patients during dengue fever**. Twenty patients had mild dengue and thirty-nine had severe dengue. Box-and-whiskers graph. The box extends from the 25^th ^to the 75^th ^percentile and the line at the middle is the median. The error bars, or whiskers extend down to the lowest value and up to the highest. Mann-Whitney U test was used to evaluate differences between blood collection time from severe and mild dengue patients and no significant difference was found (P = 0.7851). Click here for file

Additional file 2 **Time dependent cytokine levels in plasma from patients with mild and severe dengue**. Twenty patients had mild dengue and thirty-nine had severe dengue. Dots represent single patient's cytokine measurements. Click here for file
