# Supplementary figures and images for: Multiplex cytokine profile from dengue patients: MIP-1beta and IFN-gamma as predictive factors for severity
Source: BMC Infect Dis. 2008 Jun 25;8:86. doi: 10.1186/1471-2334-8-86 (PMC2474613; doi:10.1186/1471-2334-8-86)

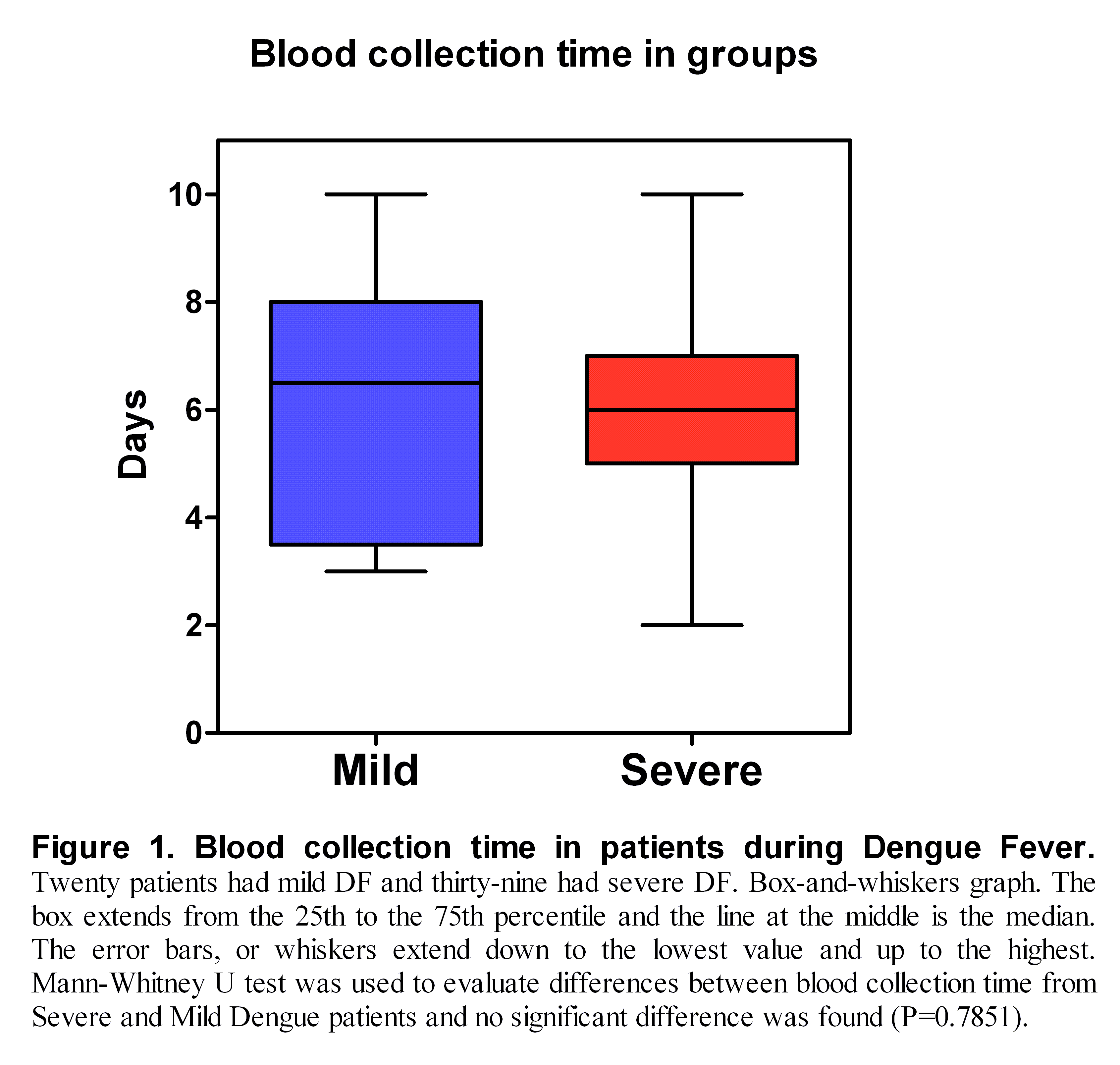

Supplement: Additional file 1 — Blood collection time in patients during dengue fever. Twenty patients had mild dengue and thirty-nine had severe dengue. Box-and-whiskers graph. The box extends from the 25th to the 75th percentile and the line at the middle is the median. The error bars, or whiskers extend down to the lowest value and up to the highest. Mann-Whitney U test was used to evaluate differences between blood collection time from severe and mild dengue patients and no significant difference was found (P = 0.7851). [file 1471-2334-8-86-S1.bmp]

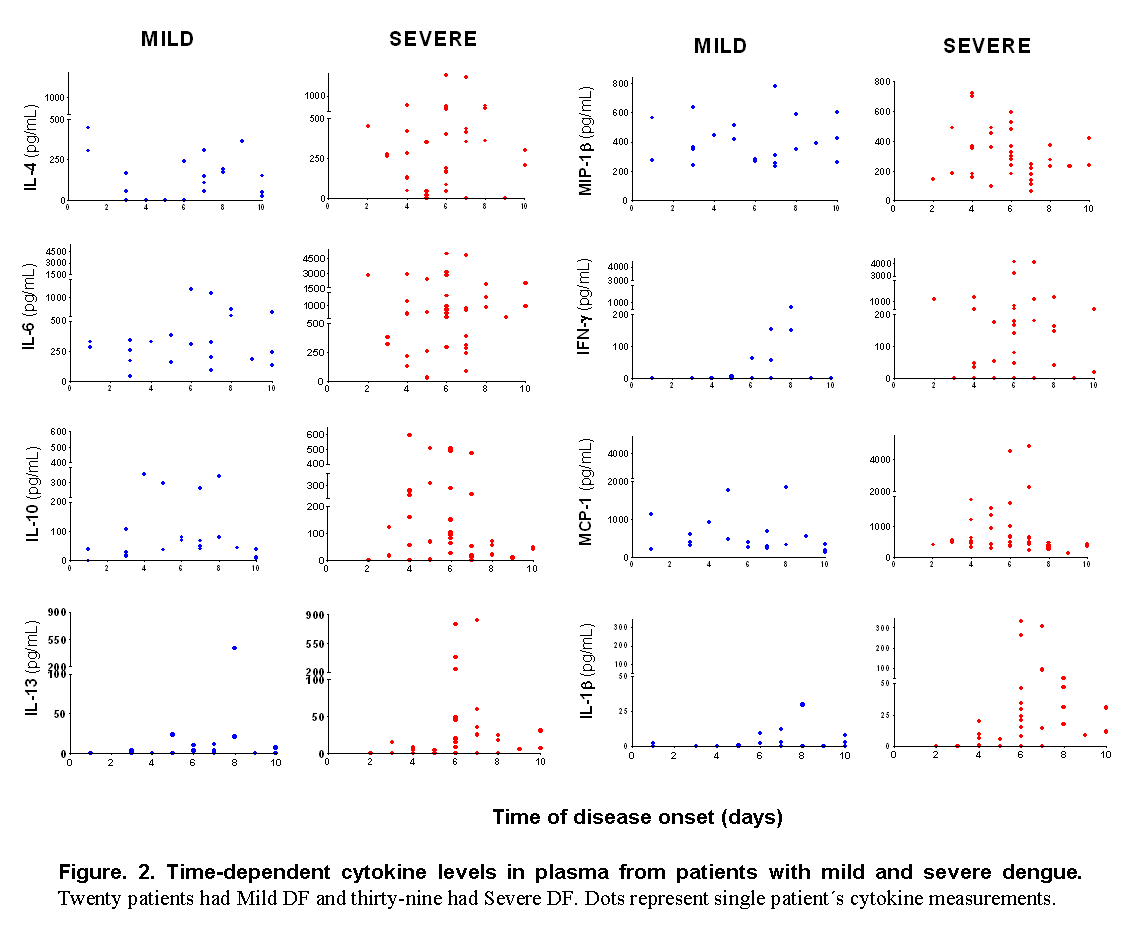

Supplement: Additional file 2 — Time dependent cytokine levels in plasma from patients with mild and severe dengue. Twenty patients had mild dengue and thirty-nine had severe dengue. Dots represent single patient's cytokine measurements. [file 1471-2334-8-86-S2.bmp]
